# Scientific Items

**Published:** 1885-10-20

**Authors:** 


### SCIENTIFIC ITEMS.

**Is the Air Colorless?**—The Challenger has dredged from the bottom of the ocean fishes which live habitually at great depths, and whose enormous eyes tell of the correspondingly faint light which must have descended to them through the seemingly transparent water. It will not be as futile a speculation as it may at first seem, to put ourselves in the condition of creatures under the sea, and ask what the sun may appear to be to them; for, if the fish who had never risen above the ocean-floor were an intelligent being, might *he* not plausibly reason that the dim, greenish light of his heaven—which is all he has ever known—as the full splendor of the sun shining through a medium which all his experience shows is transparent?

We ourselves are, in very fact, living at the floor of a great aerial sea, whose billows roll hundreds of miles above our heads. Is it not at any rate conceivable that we may have been led into a like fallacy from judging only by what we see at the bottom? May we not, that is, have been led into the fallacy of assuming that the intervening medium above us is colorless because the light which comes through it is so?

I freely admit that all men, educated or ignorant, appear to have the evidence of their senses that the air is colorless, and that pure sunlight is white, so that if I venture to ask you to listen to considerations which have lately been brought forward to show that it is the sun which is blue, and the air really acts like an orange veil or like a sieve which picks out the blue and leaves the white, I do so in the confidence that I may appeal to you on other grounds than those I could submit to the primitive man who has his senses alone to trust to; for the educated intelligence possesses those senses equally, and in addition the ability to interpret them by the light of reason, and before this audience it is to that interpretation that I address myself.—From the "Sunlight and the Earth's Atmosphere," by Prof. S. P. Langley, in *Popular Science Monthly for September.*

**Forcite Powder.**—Among the explosives now in the American market is forcite powder, which is rapidly winning a name for itself among the older powders, and is battling for popular recognition in its claims for a front rank in efficiency and economy.

This new powder is very similar to explosive gelatine, the most powerful agent known among the explosives; it was invented by K. J. Sundstrom, a Swede, and patented in this country in 1881. It is a pasty or plastic gelatinized nitro-glycerine compound, and is composed of cellulose and niter and nitro-glycerine.

Its advantages as an explosive are stated as follows: It is five times *less* sensitive to shock than dynamite, and is that much safer; its semi-solid state permits it to be used with ease under any and all conditions; like explosive gelatine, it is impervious to water, and is thus valuable for military, naval, and submarine work; it is claimed to be, on the basis of volume, 25 to 50 per cent. stronger than dynamite, and its cost of manufacture is about the same as dynamite.

In support of these claims for the new powder we notice, says Engineering News, that Gen. Henry L. Abbot, in an official report upon the test of this powder, says that taking dynamite No. 1 as a standard, and giving it a value of 100, the forcite, with 95 per cent. equals an intensity of 133; with 75 per cent. it equals 124; and with 40 per cent. strength it equals an intensity of 95. In a personal letter to the manufacturers of the forcite, published with the report, Gen. Abbot also says: "Your explosive is the strongest to be had in our market, and must therefore be a prominent candidate for adoption in our torpedo service in place of dynamite No. 1."—*Scientific American.*

**The Minuteness of Germs.**—It is altogether beyond the power of the mind to conceive the minute size of some of the germs which, in their subsequent development, work such wondrous changes, and which have such important influences on health and several industrial processes. We read of the experiments of Pasteur, Tyndall, and others; but we seldom realize the infinitely small size of the organisms and germs referred to for some are undoubtedly so minute that the most powerful microscope fails to detect them. The minute organisms capable of inducing changes analogous to the fermentation caused by yeast, have received great attention of late years; and several important diseases are distinctly traced to them. Bechamp estimated that eight billions of germs of one micro-ferment occupied only one cubic twenty-fifth of an inch. Not one of these minute bodies could develop except by carrying on complicated processes of a chemical nature, involving very active movements of its atoms and molecules.

The mathematicians have made calculations, founded on the pressure exerted by the gases, and other considerations, which show that a particle of the sort matter, such as albumen and protoplasm, chiefly concerned in life process, contains in a space of one cubic thousandth of an inch more molecules than any one could possibly form any conception of. Sorby, taking a portable mean of such calculations, supposes one cubic thousandth of an inch of water to contain 3,700,000,000,000,000 molecules. A sheet of ordinary note-paper is about one-hundredth of an inch thick. One-tenth of this would, of course, be one-thousandth of an inch; and a little cubical box of that size each way would hold the amazing number of water molecules mentioned. Perhaps a few thousands of such molecules may suffice for some manifestations of life; but, even if many millions should be requisite for the structure of the humblest and simplest germ, we could never expect to see the actual beginning of life.—*Pop. Sci. News.*

**Indian-Ink** for drawing plans may be prevented from running by adding a little sugar to the ink.

